# Bio-Nanocomposite Hydrogel Based on Zinc Alginate/Graphene Oxide: Morphology, Structural Conformation, Thermal Behavior/Degradation, and Dielectric Properties

**DOI:** 10.3390/polym12030702

**Published:** 2020-03-22

**Authors:** Roser Sabater i Serra, José Molina-Mateo, Constantino Torregrosa-Cabanilles, Andreu Andrio-Balado, José María Meseguer Dueñas, Ángel Serrano-Aroca

**Affiliations:** 1 Centre for Biomaterials and Tissue Engineering, Universitat Politècnica de València, 46022 València, Spain; jmmateo@fis.upv.es (J.M.-M.); ctorregr@fis.upv.es (C.T.-C.); jmmesegu@upv.es (J.M.M.D.); 2 CIBER-BBN, Biomedical Research Networking Centre in Bioengineering, Biomaterials and Nanomedicine, 46022 València, Spain; 3 Departament de Física, Universitat Jaume I, 12071 Castelló, Spain; andrio@fca.uji.es; 4 Biomaterials and Bioengineering Lab, Centro de Investigación Traslacional San Alberto Magno, Universidad Católica de Valencia San Vicente Mártir, 46001 València, Spain

**Keywords:** nanocomposite, hydrogel, alginate, graphene oxide, zinc, thermal and dielectric properties, bioactivity, biocidal effect, tissue engineering

## Abstract

Bio-nanocomposite hydrogels based on sodium alginate (SA) as polymer matrix and graphene oxide (GO) nanosheets with zinc as crosslinking agent were synthesized with the aim of incorporating the intrinsic properties of their constituents (bioactivity and antimicrobial activity). Thus, stable and highly interconnected networks were obtained from GO nanosheets dispersed in SA matrices through interactions with low amounts of zinc. The GO nanosheets were successfully incorporated into the alginate matrix in the form of a complex nano-network involving different interactions: Bonds between alginate chains induced by Zn ions (egg box structure), interactions between GO nanosheets through Zn ions and hydrogen bonds between alginate chains, and GO nanosheets. The molecular interactions and morphology were confirmed by Fourier-transform infrared spectroscopy and transmission electron microscopy. The composite’s structural organization showed enhanced thermal stability. The glass transition temperature shifted to a higher temperature due to the reduced mobility induced by additional crosslinking bonds after incorporating the GO nanosheets and Zn into the polymer matrix. Finally, the dielectric behavior revealed that charge carrier mobility was hampered by the compact structure of the nanonetwork, which reduced conductivity. The combined properties of these nanocomposite hydrogels make them attractive biomaterials in the field of regenerative medicine and wound care since both surface bioactivity and antibacterial behavior are two critical factors involved in the success of a biomaterial.

## 1. Introduction

Polymer-based nanocomposites are produced by including nanomaterials distributed in the pristine polymer matrix with at least one phase in the nanoscale dimension. The presence of the nanomaterial within the polymer matrix improves the properties, such as higher thermal stability or better mechanical performance, but can also generate a new set of properties that depend on the type of nanomaterial incorporated [1]. In this regard, carbon nanomaterials (CNMs) such as graphene and graphene oxide (GO) are rapidly emerging as a new class of fillers to produce nanocomposites with enhanced properties [2].

Graphene and its derivatives have received a great deal of attention in biomedical applications such as drug and gene delivery, imaging, and tissue engineering, due to their high conductivity, good mechanical properties, ability to induce conductivity when combined with biopolymers, and ease of processing [3]. Graphene oxide, a well-known carbon nanomaterial with sp^2^-hybridized single-atom-layer structure, has a large specific surface area, with unique physicochemical properties [4]. The multifunctional properties of graphene-based materials have been reported to enhance the cellular response of different cell lines [5]. Several studies have shown that GO promotes mesenchymal stem cell growth and differentiation toward osteogenic [6] and myogenic lineages [7]. Nanocomposites prepared from biopolymers such as polylactic acid/poly lactic-co-glycolic acid, polyacrylamide/gelatin, poly(3-hydroxybutyrate-co-3-hydroxyvalerate) together with graphene derivatives, particularly GO, have been investigated for tissue engineering applications with promising results [8,9,10,11]. GO is also considered as a biocidal nanomaterial, with strong antimicrobial activity [12,13]. GO nanosheets have recently been incorporated in calcium alginate hydrogels to improve their mechanical behavior, water diffusion, and many other physical properties [14,15,16,17], while its antibacterial capacity and negligible cytotoxicity in mammalian cells have been probed at very low GO concentrations [18].

Alginate, a natural anionic polysaccharide approved by FDA, is considered a promising biomaterial with exceptional hydrophilicity, biocompatibility, biodegradability, nontoxicity, and low cost in comparison with other materials [19]. Its excellent properties render this hydrophilic material useful in a broad range of biomedical applications such as drug delivery and immobilization of cells, enzymes, and proteins [20,21]. Alginates are commonly used as scaffold materials for cells in regenerative medicine and tissue engineering [22]. This biopolymer possesses a linear chain structure of (1–4)-linked β-d-mannuronic acid (M) and α-l-guluronic acid (G) residues arranged in block-wise fashion with M and G present in different proportions and sequences, depending on the source of the alginate [23,24]. Divalent cations such as Ca^2+^ and Zn^2+^ can interact with blocks of G residues of sodium alginate (SA) to produce alginate gels arranged according to the classical egg box model [25].

Zinc is one of the most essential biometals in the human body, as well as Fe, Cu, Mn, and Co. It is found in all body tissues, with 85% of the body’s zinc in muscle and bone [26]. It is a fundamental microelement involved in cell proliferation and differentiation and a structural constituent of a great number of proteins, including enzymes involved in cellular signaling pathways and transcription factors [27,28]. Biodegradable metals such as zinc have recently attracted considerable attention in tissue engineering [29,30] and wound healing [31]. Zinc has been proposed as a new biomaterial for use in bioabsorbable cardiac stents [32] and as a structural material for biodegradable implants [29]. The release of zinc ions from implant during the degradation phase can integrate into normal metabolic activity without producing systemic toxic effects. It has also been found to promote myogenic differentiation of myoblast [33] and osteogenic differentiation of mesenchymal stem cells [34,35]. Even though its role in numerous metabolic processes has been established [36], the viability of zinc-incorporated biomaterials in clinical applications is based on many factors, mainly safety problems related to zinc content and release kinetics. Uncontrolled release of Zn^2+^ can affect zinc homeostasis, alter the concentrations of other trace metals, and produce protein dysfunction [37]. The optimal zinc content depends on multiple factors, such as physicochemical properties of materials, cell lines, and release kinetics [33,37,38]. Altogether, the ideal solution to ensure the safety of Zn-incorporated biomaterials is to keep the amount of zinc to a minimum while maximizing its biological response.

Both surface bioactivity and antibacterial behavior are two critical factors in determining the success of a biomaterial in an in vivo environment. Thus, a new approach that combines both properties would have real potential in the field of biomedicine. In the present work we used sodium alginate as polymeric matrix and GO nanosheets with zinc as a crosslinker to synthetize new nanocomposites hydrogels that combine the properties of their constituents, bioactivity, and antimicrobial activity. The zinc concentration in the nanocomposites synthesized was restricted to the minimum amount required to achieve alginate gelation, promote bioactivity, and avoid toxic effects. The quantity of GO nanosheet, although low (3% w/w), was enough to strengthen the alginate matrix and provide the nanocomposite hydrogel with bioactive and biocidal properties. The intrinsic properties of these nanocomposite hydrogels make them attractive biomaterials for tissue engineering, particularly skeletal-muscle and bone tissue engineering and wound care.

The physicochemical properties of zinc alginate/GO nanocomposites were determined (swelling, thermal behavior/degradation, and dielectric behavior) and compared with those of sodium alginate/GO and zinc alginate to obtain detailed information on its structure. Molecular interactions and morphology aspects of the synthesized nanocomposites, analyzed by Fourier-transform infrared spectroscopy (FTIR) and transmission electron microscopy (TEM) were also included.

## 2. Materials and Methods

### 2.1. Materials

Sodium alginate (SA) (Panreac AppliChem, Darmstadt, Germany), zinc chloride (≥97.0%, Sigma-Aldrich, St. Louis, MO, USA), used as a crosslinking agent and graphene oxide nanosheets (Sigma-Aldrich, St. Louis, MO, USA) were utilized as received without further purification.

### 2.2. Synthesis

SA films, SA with 4% w/w zinc chloride, SA with 3% w/w GO nanosheets, and SA with 4% and 3% w/w of zinc chloride and GO, respectively, were prepared by solvent casting (hereafter referred to as SA, SA/Zn, SA/GO, and SA/Zn/GO, respectively). The SA films were prepared by dissolving 0.5 g of SA in 32 mL of distilled water under continuous magnetic stirring for one hour. This aqueous solution was poured into a Petri dish and left for 24 hours in an oven at 37 °C to form thin films. SA/Zn, SA/GO, and SA/Zn/GO samples were synthesized by a similar initial procedure. First, 0.5 g of SA were dissolved in 22 mL of distilled water, the required amount of zinc chloride or/and GO to obtain 4% w/w of zinc chloride, 3% w/w of GO, or both, (referred to as the SA mass), respectively, were then dissolved/dispersed in 10 mL of distilled water. This aqueous mixture with ZnCl_2_, GO, or both was added to the previous SA aqueous solution and after one hour of continuous magnetic stirring was poured into a Petri dish and left for 24 hours in an oven at 37 °C to form thin films. The synthesized films were dried at 60 ± 0.5 °C under vacuum to constant weight.

Although the GO percentage was chosen to ensure its effect on the physicochemical properties of the resulting nanocomposite films, the ZnCl_2_ percentage was determined empirically by trial and error to obtain the minimum amount of zinc atoms to keep the final mixture in a sol state and slow down the gelation process during solvent casting.

### 2.3. Characterization Techniques

#### 2.3.1. Alginate Characterization

The sodium alginate was characterized by three techniques: Size exclusion chromatography with multi angle light scattering (SEC-MALS), high performance anion-exchange chromatography with pulsed amperometric detection (HPAEC-PAD), and nuclear magnetic resonance (NMR). Molar mass and mass distribution, block length distribution, and M/G block fractions (mono, di, and triads) were determined for this biopolymer.

#### 2.3.2. Electron Microscopy

The GO nanosheets used were observed by high-resolution transmission electron microscope (HR-TEM) on a FEI Tecnai G2 F20 (Hillsboro, Oregon, USA) 200 kV apparatus. The HR-TEM samples were prepared by dispersing the GO powder in an ultrasound bath for 10 minutes. One drop was then placed on a TEM grid with 300 mesh coated in carbon film for 10 minutes to ensure complete drying. The morphology of the four analyzed film types (SA, SA/Zn, SA/GO, and SA/Zn/GO) was observed on a JEM-1010 (JEOL, Tokyo, Japan) 100kV transmission electron microscope (TEM) at a magnification of 15,000×. The TEM samples were ultrathin sections (60 nm) sliced by a Leica Ultracut UC6 ultramicrotome and Diatome diamond knife. TEM grids with 300 mesh coated in carbon film were used for specimen deposition.

#### 2.3.3. Fourier-Transformed Infrared Spectroscopy (FTIR)

The presence of specific chemical groups in the composite films was identified by FTIR at room temperature with a Bruker Optics FTIR Alpha II (Billerica, MA, USA). FTIR spectra were collected in transmittance mode from 4000 to 300 cm^−1^ after 24 scans at a resolution of 2 cm^−1^.

#### 2.3.4. Swelling Assay

The swelling experiments were performed gravimetrically in triplicate. Dry square samples (10 × 10 mm) with thickness lower than 100 microns were vacuum dried at 60 °C and immersed in water until equilibrium at room temperature (25 °C). The swelling degree, *w_eq_*, was expressed as the amount of solvent per unit mass of the dry polymer.

#### 2.3.5. Differential Scanning Calorimetry

Differential scanning calorimetry (DSC) analysis was performed on a PerkinElmer DSC 8000 (Waltham, Massachusetts, USA) under a flowing nitrogen atmosphere. The first scan was on dry samples from 0 to 300 °C at a heating rate of 20 °C/min. A second scan (cooling) was carried out from 150 to 0 °C at a rate of 10 °C/min after heating the samples at 150 °C for 10 min to remove all traces of water.

#### 2.3.6. Thermogravimetric Analysis (TGA)

Measurements were performed on a Mettler Toledo TGA 2 (small furnace (SF) system (Columbus, Ohio, USA). The samples (5–10 mg weight) were placed on the balance and the temperature raised from 30 to 900 °C at a rate of 10 °C/min. The mass of the sample was continuously monitored as a function of temperature.

#### 2.3.7. Dielectric Relaxation Spectroscopy (DRS)

Dielectric measurements were carried out on an Alpha-S impedance analyzer in the range from 0.1 Hz to 1 MHz. Samples around 0.1 mm thick were dried under vacuum and placed in a 10 mm diameter parallel plate capacitor mounted on a temperature-controlled cryostat (BDS 1100) and exposed to a heated gas stream evaporated from a liquid nitrogen Dewar. The temperature control was assured by Quatro Cryosystem from Novocontrol GmbH (Montabaur, Germany). Isothermal scans were performed from 0 to 180 °C (thermal stability: 0.5 °C) in 5 °C steps. The complex permittivity ε* = ε′ + iε″ and complex conductivity σ* = σ′ + i σ″ were determined as a function of frequency.

## 3. Results and Discussion

### 3.1. Alginate Characterization

Sodium alginate possesses a weight-average and a number-average molecular weight of 379.5 ± 9.5 and 170.7 ± 3.1 KDa, respectively. It contains 43% guluronic acid content with 27% and 23% blocks of dimers and trimmers, respectively.

### 3.2. Electron Microscopy

**Figure 1:**
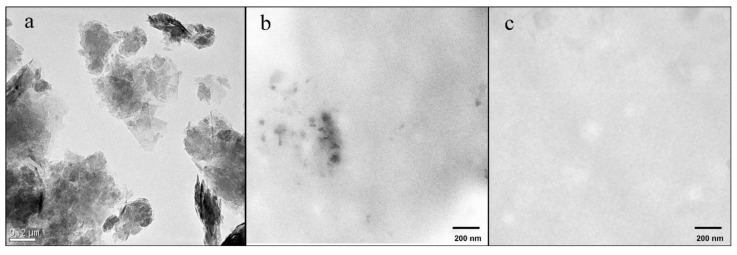
(**a**) High-resolution transmission electron microscopy of graphene oxide (GO) nanosheets; (**b**) transmission electron microscope of sodium alginate (SA) (clear phase) with 4% and 3% weight/weight (w/w) (referring to the mass of SA) of ZnCl_2_ and GO (dark phase), respectively; (**c**) transmission electron microscope of SA (clear phase) with 4% w/w (referring to the mass of sodium alginate) of ZnCl_2_.

The GO powder used in this study presented a morphology of approximately 200–400-nm-long nanosheets stacked together by van der Waals forces and π-π interactions, as shown in HR-TEM images (Figure 1a). The morphology of SA/Zn/GO composite films (TEM images) showed that these GO nanosheets (dark phase) were tightly embedded in the alginate polymer matrix (clear phase) according to their weight ratio (3/97) and thus quite difficult to observe (Figure 1b). It is of note that only some parts of the GO nanosheets (see scale bar of Figure 1b) can be appreciated in some parts of the composite sample. Similar biphasic morphology was observed for SA/GO (TEM image not shown). However, monophasic morphologies were observed for SA/Zn (Figure 1c) and SA, with only a clear phase as expected.

### 3.3. Fourier-Transform Infrared Spectroscopy (FTIR)

**Figure 2:**
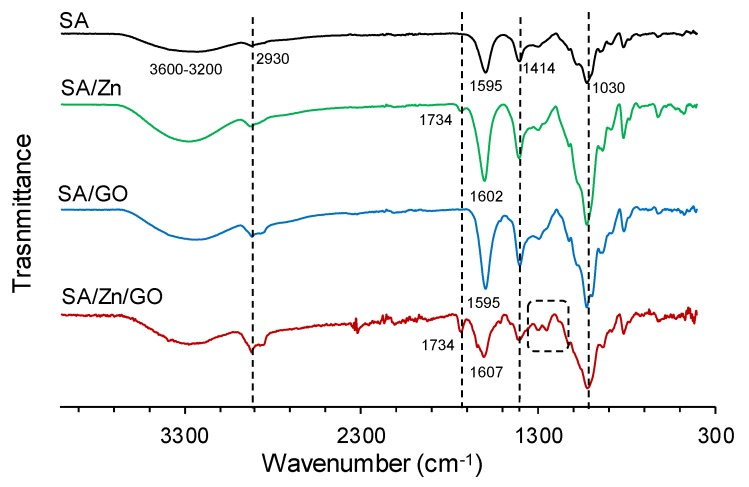
FTIR spectra of neat SA, SA/Zn, SA/GO, and SA/Zn/GO in the region of 4000–300 cm^−1^.

FTIR spectra of all the samples are shown in Figure 2. For native sodium alginate, the absorption peaks were mainly assignable to O–H stretching vibration at 3200–3600 cm^−1^, with a peak at 3226 cm^−1^ [39]. The peak at 2930 cm^−1^ can be ascribed to overlapping symmetric and asymmetric C–H stretching vibration of aliphatic chains [40] and the band at 1030 was assigned to the stretching vibration of C–O–C groups [41]. The peaks at 1595 and 1414 cm^−1^ corresponded to symmetric and asymmetric carbonyl stretching vibration of carboxylate salt group [42]. The carbonyl stretching shifted from 1595 shifts to 1602 cm^−1^ after Zn crosslinking, which can be related to the formation of coordinate bonds between the carboxylate moieties of alginic acid and Zn ions, increasing the double bond of carboxylic carbonyl that produced the shift to higher stretching energies [39]. The SA/Zn/GO composites showed an additional shift of the peak related to carbonyl stretching, from 1595 to 1607 cm^−1^, suggesting further interaction between SA chains and GO nanosheets though Zn ions. An additional vibrational band, about 1734 cm^−1^, was found only in samples containing Zn ions (SA/Zn and SA/Zn/GO). The band, assigned to C=O stretching [43], may also be attributed to the crosslinking effect of Zn.

With GO incorporation (SA/GO and SA/Zn/GO nanocomposites), the peak in the band 3600–3200 cm^−1^ (O-H stretching vibration) shifted slightly to lower wave numbers and broadened, which may be attributed to the alginate-GO interaction though intermolecular hydrogen bonds. FTIR spectra indicated that sodium alginate and GO were strongly intertwined by the hydrogen bonds between oxygen-containing groups in alginate chains and GO nanosheets [44]. Hydrogen bonding through electrostatic attraction may induce good interfacial adhesion at the SA/GO interface [42]. The strong peaks in the band at 1000–1200 cm^−1^, assigned to the C–O and C–O–C stretch, as mentioned before, were also observed when carboxylic acid groups interacted with divalent ions [17,45], which may lead to the crosslinking of alginate chains and GO nanosheets.

**Figure 3:**
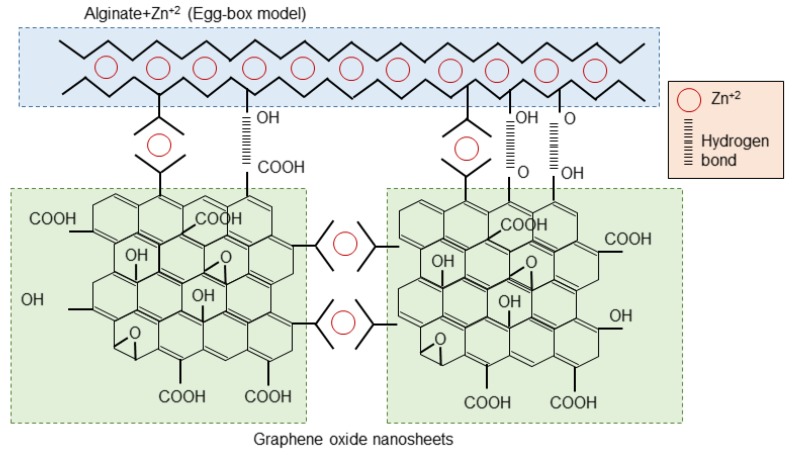
Schematic model of SA/Zn/GO nanocomposite.

It has been reported that multivalent ions, particularly Ca^2+^, Ba^2+^, and Fe^3+^ are able to crosslink both alginate and GO nanosheets to form crosslinked GO networks inside the hydrogels of alginate [17,44]. In this study, despite their low concentration, divalent Zn ions were able to interact synergistically with both SA chains and GO during the gelation process. The structure of this complex nano-network, which involved all three constituents of the composite, is depicted in Figure 3. Three types of interactions were involved in the structure of SA/Zn/GO nanocomposites: Bonds between SA chains induced by Zn ions (egg box structure), interactions between GO nanosheets through Zn ions, mainly by carboxylic groups [46], and hydrogen bonds between SA chains and GO nanosheets through hydroxyl, carboxylic, and carbonyl reactive groups.

### 3.4. Swelling Behavior

Water sorption was determined gravimetrically by obtaining the amount of water absorbed by the sample from the dry to the swollen state in the crosslinked samples (SA/Zn and SA/Zn/GO). SA/GO nanocomposites, although both components interact with each other through hydrogen bonds (FTIR results), dissolved after immersion in water [2], indicating that the links were not strong enough to prevent the dissolution of the alginate chains. SA samples crosslinked with zinc ions produced networks with a very high swelling degree in equilibrium (*w_eq_* ca. 116) due to their low crosslinking density produced by the low percentage of zinc chosen. These hydrogels show low stability and after immersion in water for three days, signs of degradation were apparent with loss of structural integrity. However, the composites synthesised after GO incorporation together with zinc ions (SA/Zn/GO sample) produced hydrogels with good structural integrity after immersion in water and a high degree of swelling (*w_eq_* ca. 88), even though water sorption was less than in the SA/Zn networks (Table 1). The hydrogel structure remained after immersion in water for two weeks.

These results indicated an increase in the crosslinking density in SA/Zn/GO nanocomposites, suggesting the presence of additional bonds within the composite after GO incorporation, consistent with the schematic model in Figure 3.

### 3.5. Thermal Properties

Thermal behavior and degradation were analyzed by differential scanning calorimetry and thermogravimetry, respectively, to get further insight into the nanocomposites’ molecular arrangement and validate the proposed structure shown in Figure 3.

#### 3.5.1. Differential Scanning Calorimetry

**Figure 4:**
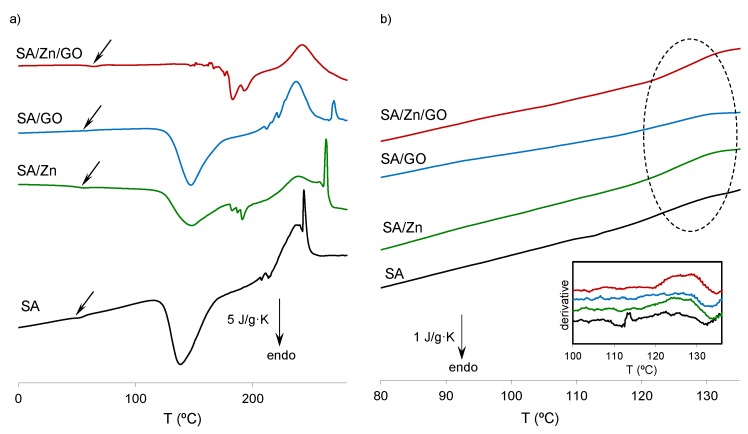
(**a**) Normalized heat flow on heating at 20 °C/min, (**b**) normalized heat flow on cooling at 10 °C/min after dehydration. The inset in (**b**) shows the temperature derivative of the normalized heat flow from 100 to 140 °C.

Figure 4a shows the normalized heat flow after synthesis (without erasing the thermal history). A first, small endotherm peak can be seen in the interval between 50 and 70 °C, followed by a larger one at 115 °C and 180 °C in the neat SA, SA/Zn, and SA/GO samples. Polysaccharides, including alginates, have a strong affinity for water and their hydration properties depend on their molecular structure [47]. Alginates release water at different temperatures depending on the different interaction between water-alginate chains: Free water, released in the 40–60 °C interval; water linked through hydrogen bonds, released in the region of 80–120 °C; and more tightly linked water through polar interactions with carboxyl groups at a higher temperature (up to 160 °C) [47,48]. The samples were vacuum-dried at 60 °C for 24 h. However, a very small endothermic peak can be seen in all of them in the 50–65 °C interval (see arrow in Figure 4a), which can be related to the release of free water. The second endotherm peak in the thermogram (interval 120–170 °C) may be related to water tightly bonded through polar interactions with carboxylate groups. It is noteworthy that the largest peak was in the SA sample, followed by the SA/GO nanocomposite. Both samples possessed –COOH groups that can interact with water molecules; in addition, GO and SA can interact with each other though hydrogen bonds, as shown in Figure 3. The peak area in SA/GO nanocomposite, smaller than neat SA, as stated above, suggests that a part of the reactive bonds interacted with water molecules, although there were also interactions between GO nanosheets and SA chains, which resulted in less water bonded. When SA was crosslinked with Zn ions (by bonding the carboxylate groups of the guluronate groups on the polymer backbone) [49], and taking into account the small amount of Zn^2+^ incorporated, the carboxylate reactive groups that remained after crosslinking were able to link to water molecules, but to a lesser extent, so that the amount of linked water molecules decreased, as can be seen in the SA/Zn thermogram. The SA/Zn/GO nanocomposite did not show any peak in this interval, indicating strong links between the SA chains, GO nanosheets, and Zn ions, which resulted in a high crosslinked network with few free reactive groups able to bind to water molecules.

SA matrix degradation took place at temperatures above 180 °C, in good agreement with the TGA results described below. The SA/Zn and SA/Zn/GO samples showed an irregular peak in the 180–200 °C interval. This irregular peak was also noticeable with a smaller amplitude in the thermal profile of neat SA and SA/GO composite, although at higher temperatures (ca. 200–210 °C). The shift to a lower temperature was related to zinc-induced degradation of alginate. Zn^2+^ is an effective Lewis acid able to coordinate hydroxyl groups and cleave to C–O bonds, particularly at high temperatures [50,51]. A broad exotherm peak at temperatures higher than 220 °C was observed in all the samples, related to the full degradation process, with maximum thermal decomposition occurring at 240–245 °C.

A second scan (on cooling) was carried out after heating the samples at 150 °C to remove all traces of water (Figure 4b and inset). The glass transition process can be now seen in all the samples. The glass transition temperature, *T_g_*, the width of the glass transition, Δ*T_g_* and the heat increment at the glass transition, Δ*c_p_*, are summarised in Table 1. As expected, *T_g_* increased with the incorporation of crosslinking points induced by Zn^2+^ ions (SA/Zn sample) because of the reduced mobility of the chain segments. With further crosslinking density that led to loss of mobility, SA/Zn/GO nanocomposites showed the highest *T_g_.* Since *T_g_* represents the onset of cooperative segmental motions, increasing crosslinking density reduced long-range chain movements, more energy was required to induce segmental motions, and *T_g_* rose. On the other hand, in the SA/GO nanocomposite, which was not crosslinked, *T_g_* remained at the same temperature as neat SA. The Δ*c_p_* decreased in the nanocomposites, particularly in the SA/Zn/GO nanocomposite, denoting that molecular mobility diminished as the network became more crosslinked. The Δ*T_g_*, related to the distribution of mobility of the polymer segments, was higher than neat SA, suggesting a structural heterogeneity that could be attributed to the existence of domains with different mobility [52,53].

#### 3.5.2. Thermogravimetry Analysis

**Figure 5:**
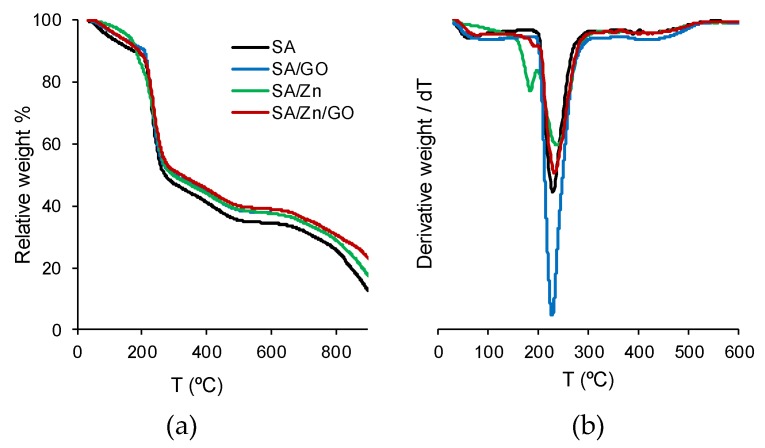
Thermogravimetry results. (**a**) Relative weight loss, (**b**) temperature derivative of weight loss vs. temperature.

The thermogravimetry results and derivative TGA curves are represented in Figure 5a,b. Neat SA exhibited an initial loss of weight due to the removal of water molecules (free and bound water at temperatures lower than 180 °C), in good agreement with the differential scanning calorimetry (DCS) results. The derivative showed a first peak in Zn-containing samples (SA/Zn and SA/Zn/GO) around 185 °C, related to Zn-induced degradation of the alginate matrix, also in good agreement with the DCS results (Figure 4a). The maximal thermal decomposition occurred in the 210–290 °C interval (peak ca. 240 °C in Figure 5b), also found in the DSC thermograms, followed by a second degradation in the 290–550 °C interval, and a final decomposition at temperatures higher than 600 °C. The first and second steps of rapid degradation could be attributed to SA matrix degradation, while the last process, at a much higher temperature, could be related to the formation of metal carbonates [54]. A slight shift to higher temperature can be seen in SA/Zn/GO nanocomposites at temperatures above 240 °C. At higher temperatures, the addition of Zn ions, GO nanosheets, or both reduced the weight loss. The temperature at which the weight loss was 50%, *T*_*d*-50%_, was about 50 °C higher in the SA/Zn/GO nanocomposite than neat SA (Table 1). In addition, the residual weight at 900 °C of samples that contained Zn^2+^ or GO was higher than that of neat SA, with maximum values for the SA/Zn/GO nanocomposite. It can, therefore, be concluded that thermal stability increased after adding both Zn ions and GO nanosheets to the SA matrix.

### 3.6. Dielectric Properties

**Figure 6:**
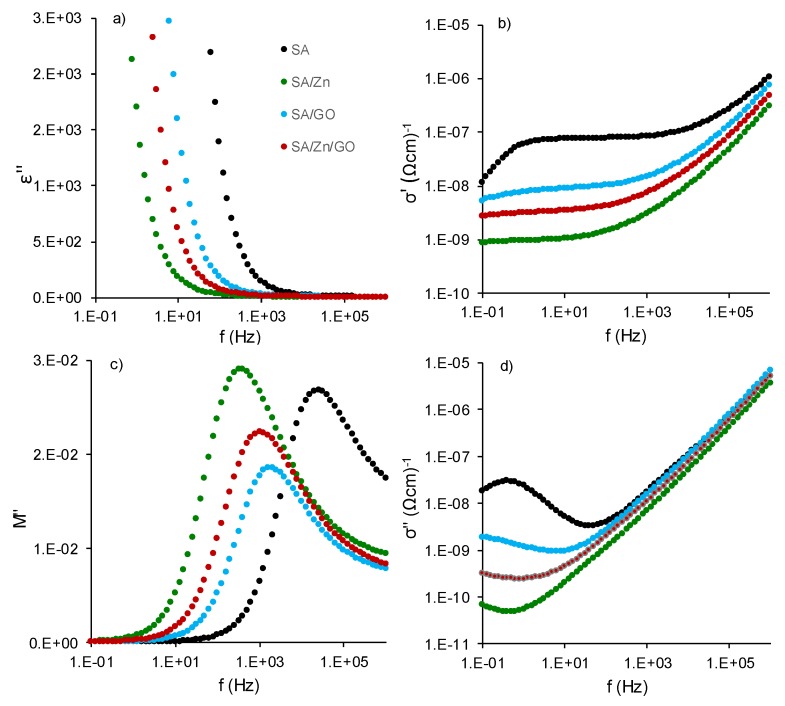
Frequency dependence obtained at 20 °C from the dielectric relaxation spectroscopy (DRS) spectra. (**a**) Imaginary part of the permittivity; (**b**) real part of the conductivity, (**c**) imaginary part of the electric modulus, (**d**) imaginary part of the conductivity.

The ability of SA to form complex structures with Zn ions and GO nanosheets was assessed using broadband dielectric relaxation spectroscopy to study molecular mobility and structural transitions in the nanocomposites. Figure 6a shows the isotherm curves of the imaginary part of the dielectric permittivity, *ε*″, at 20 °C, for all the samples. The high value of the dielectric loss, *ε*″ at low frequencies, with no relaxation process observed, was related to conductivity. Depending on the material, the conductivity can be due to free charges, ionic conductivity, interfacial polarization (the so-called Maxwell–Wagner–Sillars (MWS) effect), and electrode polarization [55,56]. High values were also found for the real part of the conductivity, *ε*′ (results not shown). This behavior could be related to MWS polarization and electrode polarization, which can result in high values of the real part of dielectric permittivity, ε^*^, in addition to the dielectric loss from the so-called direct current (dc) conductivity [55]. The MWS effect occurs in polymer composites due to the migration of mobile charges under the influence of an electric field accumulating at the interface of the constituents [57]. To gain further insight into the effect of the conduction behavior (charge carrier movement, MWS effect) and its associated relaxation, the complex conductivity, σ^*^ together with the electric modulus formalist M^*^ has been used in complex materials, such polymer composites [57].

The complex conductivity can be calculated by Equation (1):

(1) $$\sigma^{*}=\sigma^{\prime }+i\sigma^{\prime \prime }=\epsilon_{0}\omega \epsilon^{\prime \prime }\left(\omega \right)+i\epsilon_{0}\omega \epsilon^{\prime }\left(\omega \right)$$

where *ε*_0_ (8.85×10^−12^ Fm^−1^) is the permittivity of free space and *ω* = 2π*f* is the angular frequency.

**Figure 7:**
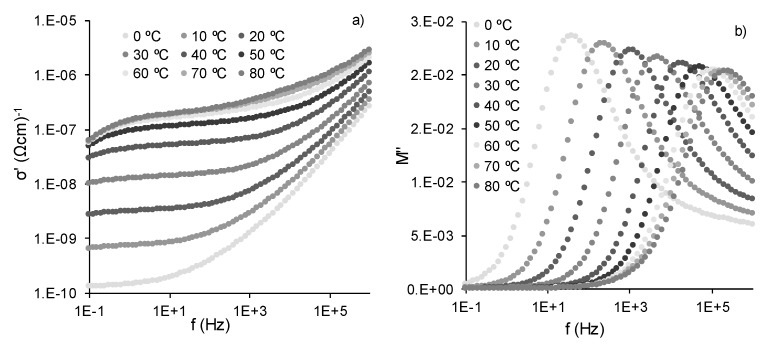
Isothermal spectrum between 0 and 80 °C for SA/Zn/GO composite. (**a**) Real part of the conductivity and (**b**) imaginary part of the electric modulus.

Figure 6b,d shows the real and imaginary part of the conductivity (*σ*′ and *σ*″) of all the samples at 20 °C. Two well-identified regimes can be seen, which depend on the frequency. For low frequencies (between 1 to 100 Hz), conductivity was nearly independent of frequency (a plateau can be seen), this being the regime dominated by dc-conductivity. The dependence of conductivity on temperature can be observed (alternating current (ac) conductivity) at higher frequencies (>10^3^ Hz). An additional feature can be identified at very low frequencies (below 10 Hz), particularly in neat SA, where conductivity decreased. This phenomenon, also observed as an increment (peak) in the *σ*″ spectra, can be ascribed to space charge polarization at the blocking electrode (electrode polarization) [55,58]. The real part of the conductivity for SA/Zn/GO nanocomposites from 0 to 80 °C is depicted in Figure 7a, where dc- and ac-conductivity can be identified, shifting to high frequency with increasing temperature, as well as electrode polarization for temperatures over 30 °C.

Figure 6b shows that the real part of the conductivity for neat SA was the highest, suggesting that the mobility of the charges was reduced due to the interactions between Zn^2+^ with SA chains (SA/Zn sample), GO nanosheets (SA/GO nanocomposite), or both, in the case of the SA/Zn/GO nanocomposite. Comparable results were obtained in which the conductivity of SA crosslinked films was less than neat SA after the interaction between the SA chains with the calcium ions (also divalent as zinc ions) [47] and GO nanosheets [2]. At 20 °C, conductivity in SA/GO nanocomposites was lower than pure SA, due to the high insulating properties of the GO nanosheets [46]. Crosslinked nanocomposites (SA/Zn and SA/Zn/GO) showed the lowest values, indicating that crosslinking led to a more compact structure, so that the mobility of the carriers was constrained.

**Figure 8:**
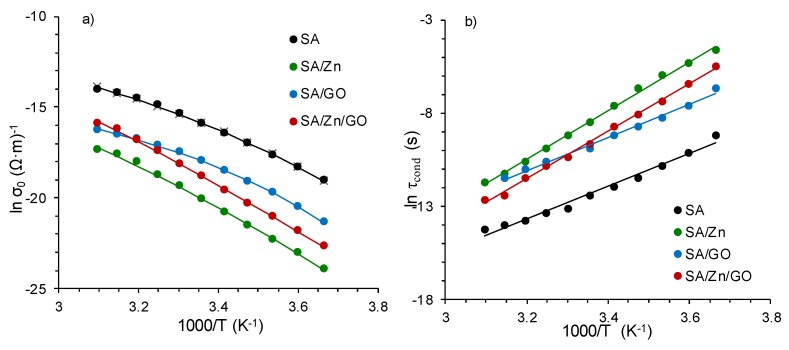
(**a**) Temperature dependence of dc-conductivity, *σ*_0_, obtained from *σ*′ from 0 to 50 °C; (**b**) temperature dependence of the relaxation time related to the conductivity process, *τ_cond_*, in the same temperature range. Solid lines show VFT (**a**) and Arrhenius (**b**) fitting.

With regard to conductivity dependence on temperature, Figure 8a shows that *σ*_0_, related to dc-conductivity (values from the plateau in *σ*′), increased with temperature similarly in all the samples, which reflected the mechanism of charge transport of carriers. The temperature dependence of dc-conductivity exhibited a Vogel–Fulcher–Tammann (VFT) relation, characteristic for glass-forming liquids:

(2) $$\sigma_{0\;}\left(T\right)=\sigma_{\infty }e^{\frac{B}{T-T_{0}}}$$

where *σ_∞_* is the dc-conductivity for the infinite temperature, *B* is a constant, and *T*_0_ is the Vogel temperature.

The solid lines in Figure 8a show the good agreement between experimental results and VFT fitting. The parameters included in Table 2 show that *T*_0_ for the crosslinked samples (SA/Zn and SA/Zn/GO nanocomposite) was lower than the neat SA and SA/GO nanocomposite. The transition from the frequency-independent (dc-conductivity) to the frequency-dependent regime (ac-conductivity) signalled the onset of the conductivity relaxation phenomenon.

The electric modulus formalism M^*^, besides being a useful tool for analyzing the dielectrical properties in composite materials [57], limits the effect of electrode polarization. It is defined as the reciprocal of the complex relative permittivity, ε^*^:

(3) $$M^{*}\left(\omega \right)=\frac{1}{\varepsilon^{*}}=M^{\prime }+iM^{\prime \prime }=\frac{\epsilon^{\prime }\left(\omega \right)}{\varepsilon^{{}^{\prime }2}\left(\omega \right)+\varepsilon^{{}^{\prime \prime }2}\left(\omega \right)}+i\frac{\epsilon^{\prime \prime }\left(\omega \right)}{\varepsilon^{{}^{\prime }2}\left(\omega \right)+\varepsilon^{{}^{\prime \prime }2}\left(\omega \right)}$$

where *M*′ and *M*″ are the real and imaginary part of the complex electric modulus, respectively, and *ω* is the angular frequency.

Figure 6b shows *M*″ isotherms at 20 °C of all the samples, in which a peak can be seen in the same range as the conductivity phenomenon (*ε*″ spectrum in Figure 6a). Figure 7b shows the *M*″ spectrum of the SA/Zn/GO nanocomposite at several temperatures. The *M*″ peak position, shifting to higher frequencies with increasing temperature, was in the transition region from dc- to ac-conductivity, as can be seen in Figure 7a,b. The *M*′ and *M*″ spectra of the other samples were similar, except for the difference in magnitudes and the peak position. The frequency of the peak (*f_max_)* was assumed to represent a characteristic frequency or relaxation time of the so-called conductivity relaxation. In this transition region (*f_max_*), charge carriers changed from long-range to short-range mobility along conducting paths [55].

The temperature dependence of the conductivity relaxation time (*τ_cond_* = 1/2π*f_max_*), obtained from the peak in the *M*″ spectrum, is represented in Figure 8b. The relaxation time decreased with increasing temperature, due to charge carrier mobility enhancement at higher temperatures [55]. Relaxation time as a function of the inverse of temperature exhibits an Arrhenius-type behavior, expressed as:

(4) $$\tau_{cond}=\tau_{0}e^{\frac{E_{a}}{k_{B}T}}$$

where *E_a_* is the activation energy of the process, *τ*_0_ is the pre-exponential factor, and *k_B_* is the Boltzmann constant.

The fitting parameters are collected in Table 2. Activation energy was the highest for the SA/Zn/GO nanocomposites, followed by zinc-crosslinked SA. The neat SA and SA/GO composite showed activation energies ca. 30% lower. This behavior appeared to be the consequence of the reduced mobility of the charge carriers due to interactions between reactive groups. Because of the more compact structure that restricts charge carrier relaxation, higher activation energy was needed in the crosslinked samples, particularly in the SA/Zn/GO nanocomposites. These results reinforced the proposed structure of the SA/Zn/GO nanocomposite synthesised in this study (Figure 3).

## 4. Conclusions

A study was made of the morphological and structural conformation, swelling, thermal, and dielectric properties of zinc alginate/graphene oxide nanocomposite networks synthesized with low amounts of zinc ions as crosslinking agent and GO nanosheets dispersed in the polymer matrix. In good agreement with the FTIR spectra, the swelling experiments suggested that the dispersed GO nanosheets were successfully incorporated into the alginate matrix in the form of a complex nanonetwork in which three types of interactions were involved: Bonds between alginate chains induced by Zn ions (egg box structure), interactions between GO nanosheets through Zn ions and hydrogen bonds between alginate chains, and GO nanosheets. Zn ions, despite their low concentration, were able to interact synergistically with both SA chains and GO during the gelation process.

The glass transition temperature shifted to a higher temperature (above zinc-crosslinked alginate networks) due to the hindered mobility induced by additional crosslinking bonds after incorporating GO nanosheets, while the molecular organization of the composite showed enhanced thermal stability. The dielectric spectrum was dominated by ionic conductivity and interfacial polarization (MWS effect). Charge carrier mobility was hindered by the compact structure produced by crosslinking, reducing conductivity and leading to higher activation energy to trigger the process.

This study clearly indicated that stable and highly interconnected nanocomposite networks can be obtained from GO nanosheets dispersed in SA matrices through interactions with low amounts of zinc.

## Figures and Tables

**Table 1:** Swelling degree in equilibrium (*w_eq_*), glass transition temperature (*T_g_*), width of the glass transition (∆*T_g_*), heat capacity increment at the glass transition (∆*c_p_*), and decomposition temperature at which weight loss was 50% (*T_d-*50%*_*).

Sample	*w_eq_* m_water_/m_dry sample_	*T_g_* (°C)	Δ*T_g_* (°C)	Δ*c_p_* (J/gK)	*T*_*d*-50%_ (°C)
SA	-	122 ± 1	5.7 ± 0.5	8.4 ± 0.002	270
SA/Zn	115 ± 2	124 ± 1	9.1 ± 0.5	7.8 ± 0.002	295
SA/GO	-	122 ± 1	9.4 ± 0.5	6.1 ± 0.002	300
SA/Zn/GO	87 ± 13	128 ± 1	8.4 ± 0.5	5.7 ± 0.002	321

**Table 2:** VFT fitting obtained for dc-conductivity, *σ*_0_, obtained from *σ*’ spectrum. Pre-exponential factor (*τ*_0_) and activation energy for the conductivity process (*E_a_*). Temperature interval for both fittings: 0 to 50 °C.

Sample	*σ*_∞_ (Ωcm^−1^)	*B* (K)	*T*_0_ (K)	*τ*_0_ (K)	*E_a_* (KJmol^−1^)
SA	3.47 × 10^−3^	−1062	1.94 × 10^2^	5.43 × 10^−19^	74
SA/Zn	2.23	−3288	1.40 × 10^2^	1.85 × 10^−23^	105
SA/GO	2.33 × 10^−5^	−573	2.19 × 10^2^	1.15 × 10^−17^	75
SA/ZN/GO	4.31 × 10^3^	−5381	9.98 × 10	1.99 × 10^−23^	106
